# The residual stress in as-built Laser Powder Bed Fusion IN718 alloy as a consequence of the scanning strategy induced microstructure

**DOI:** 10.1038/s41598-020-71112-9

**Published:** 2020-09-04

**Authors:** Itziar Serrano-Munoz, Tatiana Mishurova, Tobias Thiede, Maximilian Sprengel, Arne Kromm, Naresh Nadammal, Gert Nolze, Romeo Saliwan-Neumann, Alexander Evans, Giovanni Bruno

**Affiliations:** 1 Bundesanstalt für Materialforschung und -prüfung, Unter den Eichen 87, 12205 Berlin, Germany; 2 Institute of Physics and Astronomy, University of Potsdam, Karl-Liebknecht-Straße 24/25, 14476 Potsdam, Germany

**Keywords:** Metals and alloys, Mechanical properties

## Abstract

The effect of two types of scanning strategies on the grain structure and build-up of Residual Stress (RS) has been investigated in an as-built IN718 alloy produced by Laser Powder Bed Fusion (LPBF). The RS state has been investigated by X-ray diffraction techniques. The microstructural characterization was performed principally by Electron Backscatter Diffraction (EBSD), where the application of a post-measurement refinement technique enables small misorientations (< 2°) to be resolved. Kernel average misorientation (KAM) distributions indicate that preferably oriented columnar grains contain higher levels of misorientation, when compared to elongated grains with lower texture. The KAM distributions combined with X-ray diffraction stress maps infer that the increased misorientation is induced via plastic deformation driven by the thermal stresses, acting to self-relieve stress. The possibility of obtaining lower RS states in the build direction as a consequence of the influence of the microstructure should be considered when envisaging scanning strategies aimed at the mitigation of RS.

## Introduction

IN718 is a Ni-based superalloy widely used in aviation (e.g*.,* aircraft engines) and energy industries (e.g*.,* power generation turbines) due to its exceptional combination of high-temperature mechanical stability (up to 650 °C), good fatigue life and high resistance to degradation in corrosive or oxidizing environments^1^. Nonetheless, nickel alloys are also known for their poor machinability^2^. Compared to conventional subtractive routes, Additive Manufacturing (AM) permits the production of 3D complex near-net-shape components with greatly reduced machining, buy-to-fly ratios close to 1 and shorter production times, while also providing comparable material performances^3^. Among AM techniques, Laser Powder Bed Fusion (LPBF) consists of a layer-by-layer selective melt of a powder bed using a focused energy source. Unfortunately, one important shortcoming of LPBF is the formation of complex Residual Stress (RS) states, with values in the range of the yield strength of a material when connected to the baseplate. This RS state results from the steep spatial and temporal thermal gradients, locally induced by the laser during processing^4,5^.

RS is defined as the stress in a body with uniform temperature that is in equilibrium in the absence of external forces^6^. In general, RS is undesirable, especially if tensile, as they can negatively affect mechanical properties, distort the design geometry, and induce cracking or delamination^5,7^. Considerable amount of work (both experimental and numerical) has been reported to elucidate and mitigate macroscopic RS in AM^3^. In general, the RS tends to be tensile towards the surfaces of the sample and compressive at the centre of LPBF parts^5,8–22^. Moreover, the largest tensile values tend to occur near the top (last added layers) of the sample, when it remains connected to the baseplate. The magnitude and trend of RS have been shown to vary depending on parameters such as the geometry of the part, the material properties, and the processing parameters. In particular, it has been reported that the reduced temperature gradients, induced by shorter scan paths, decrease the magnitude of the RS^14^. Also, alternating the scan direction along the Build Direction (BD) avoids laser-path repetition, therefore increasing the homogeneity of RS fields^23^.

The speed and power of the laser affect the thermal gradient *G* (K/m) and liquid–solid interface velocity *v *(m/s) of the melt pool. These latter parameters control the solidification process and can be regulated in order to induce epitaxial (usually leading to columnar grains spanning over multiple layers) or equiaxed (the grains are randomly oriented and have a size comparable to the layer thickness) grain growth^24,25^. In the case of IN718 alloys, characteristic epitaxial columnar grains with strong <001> texture parallel to the BD have been observed by numerous authors^20,26–31^. These features result from the combined effect of a heat flux that is strongly directed toward the baseplate and of the tendency of cubic crystals to grow with <001> parallel to the heat flux^24,25^. Such strong texture has been reported to induce macroscopic mechanical anisotropy^32^.

The production of structurally reliable AM components requires further elucidation of the processing and microstructural mechanisms involved in the formation of RS. This work focuses on understanding the difference in RS and microstructure (i.e., grain size, morphology, and orientation) induced by different scanning strategies (namely, Y-scan and Rot-scan, see the section Materials and Methods). The subsurface/surface RS state is characterized using Synchrotron X-ray Energy Dispersive Diffraction (SXEDD) and Laboratory angular dispersive X-ray diffraction (LXADD). The influence of the microstructure on the build-up of RS has been examined using different Scanning Electron Microscopic (SEM) techniques, in particular Electron Back Scattered Diffraction (EBSD).

## Results

**Figure 1:**
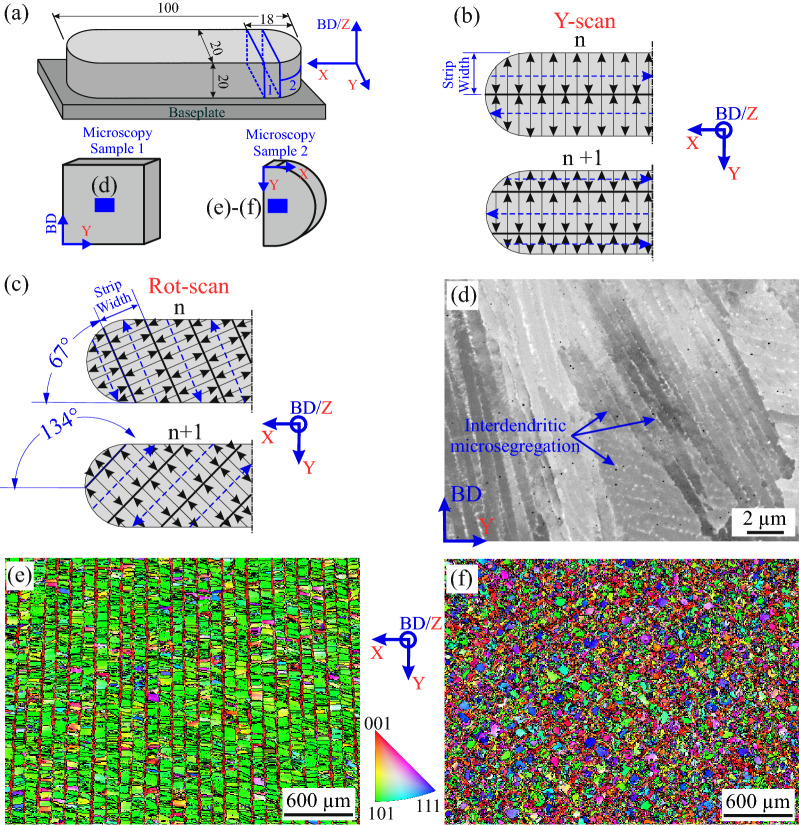
(**a**) The geometry of the samples showing the location of the microscopy samples, as well as the location of the microscopic observations. (**b**) Schematic illustration of the scanning strategies denoted as Y-scan and (**c**) Rot-scan. On these two illustrations, the Strip Width offset is shown with dashed blue lines. (**d**) High magnification Back-scattered Electron (BSE) image of as-built dendritic-cells showing columnar and mosaic structures. (**e**) Low magnification crystal orientation maps with respect to the normal direction in the EBSD measurement coordinate system (i.e., BD/Z-direction) are shown for Y-scan and (**f**) Rot-scan. The grain boundaries correspond to a misorientation angle of ≥ 5°.

Two types of bi-directional stripe-hatching strategies were studied. In the scanning strategy denoted as Y-scan (see Fig. 1a,b), the scan vector was parallel to the Y axis and the Scanning Direction (SD) was parallel to the X-direction (corresponding to the longest dimension of the sample). The stripe-hatching was performed along the Y-direction, with a Strip Width (SW) of half the width (W/2) of the sample and W/4 offset on consecutive layers. In the second scanning strategy (denoted as Rot-scan, Fig. 1c), the direction of scan vectors was incrementally rotated at each layer by 67° around the Build Direction (BD/Z-direction), while the length of the scan vectors (i.e., the stripe width) was kept identical to that of the Y-scan.

The specimens for microstructural characterization were produced by removing ~ 10 mm long cuts out of one end of the samples (see Fig. 1a). The sample denoted as Microscopy Sample 1 (MS1) was used for analysis of the BD-Y plane while the plane perpendicular to the BD (X–Y plane) was analyzed using the Microscopy Sample 2 (MS2). Figure 1d shows the dendritic-cell microstructure developed during the solidification process, where inhomogeneities of chemical composition (i.e., micro-segregation) occur along subgrain boundaries. In this figure, most of the dendritic-cells exhibit columnar structures (the micro-segregation appears as lighter lines) that tend to be generally aligned with the Z-direction (± 30° misorientation). Some mosaic structures (the micro-segregation exhibits a square-like arrangement) are also observed. Note that Fig. 1d is a local case and a detailed examination of the dendritic-cell morphology over the entire cross-sections of Y-scan and Rot-scan materials (using Microscopy Sample 1) indicate a more pronounced alternance between columnar and mosaic features, with the dendrite-width varying between ~ 300 and 800 nm.

Figure 1e,f. represent crystal orientation maps collected on the X–Y plane for both scan regimes. The color coding of <001>, <101>, <111> and all intermediate directions is used to describe the local crystallographic indexing along the BD. Figure 1e shows that a mosaic grain structure is formed in the Y-scan sample, where the scanning pattern (along Y-direction) is clearly noticeable (hereafter, all EBSD orientation maps shown in figures correspond to the normal direction in the EBSD measurement coordinate system). The central part of the pattern corresponds to grains preferably grown parallel to <101>-crystal axis with respect to the BD. The borders are usually decorated by bands of pellet-like grains aligned parallel to <001>-crystal axis with respect to the BD. In the literature^30^, the shape of the green grains is described as columnar while the red grains are considered to have an equiaxial shape. At the magnification used and with a grain boundary misorientation angle of ≥ 5°, the average grain size is 61 ± 7 µm (weighted by area fraction and corresponding to the equivalent diameter of the measured area). The grain structure resulting from the Rot-scan (Fig. 1f) exhibits grains with a larger range of crystallographic orientations. The scanning tracks are less noticeable, the average grain size is reduced to 42 ± 5 µm, and the presence of pellet-like <001>-grains is harder to distinguish.

**Figure 2:**
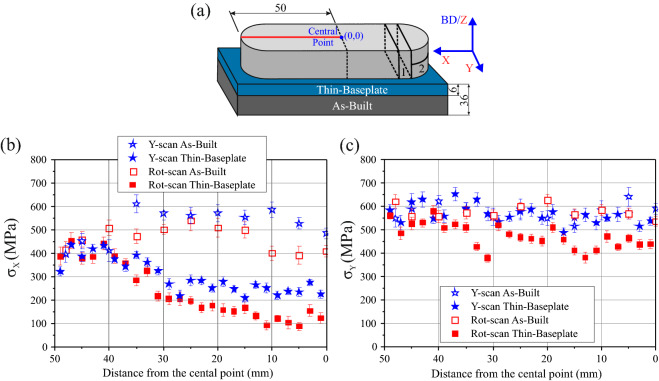
(**a**) Schematic illustration of the thinning of the baseplate showing the location of the central line where the LXADD analysis was performed**.** (**b**) Plot of the X-direction residual stress against the distance to the centre comparing the As-Built and Thin-Baseplate conditions of the two samples. (**c**) Plot of the Y-direction residual stress against distance to the centre for As-Built and Thin-Baseplate conditions.

Since the eulerian cradle used during the SXEDD experiments was not able to carry more than 5 kg, it was necessary to reduce the thickness of the baseplate from 36 to 6 mm (Fig. 2a). LXADD measurements were performed along a central line on the Top Surface of the samples in order to evaluate the effect of this baseplate thinning. In what follows, three conditions are established depending on the baseplate state: (i) As-Built condition, corresponding the state where the sample is attached to the entire baseplate, (ii) Thin-Baseplate condition, and (iii) Released condition.

As shown in Fig. 2b, the X-direction component undergoes a 250 MPa relaxation in the region between 0 and 30 mm. Interestingly, the tip region (between 40 and 50 mm) does not undergo any relaxation. On average, the Y-scan sample exhibits values that are ~ 100 MPa higher than those of the Rot-scan sample. Regarding the Y-direction, the residual stress values are similar in the As-Built condition for the two scanning strategies. After thinning of the baseplate, the residual stress values of the Y-scan sample at the tip exhibit a slight increase (~ 50 MPa), while the Rot-scan values exhibit a non-uniform relaxation (in the form of two undulations at 33 and 12 mm) of the residual stresses up to 150-200 MPa. The distortion examination of these two conditions revealed a Z-direction warping, mainly occurring at the tip region.

**Figure 3:**
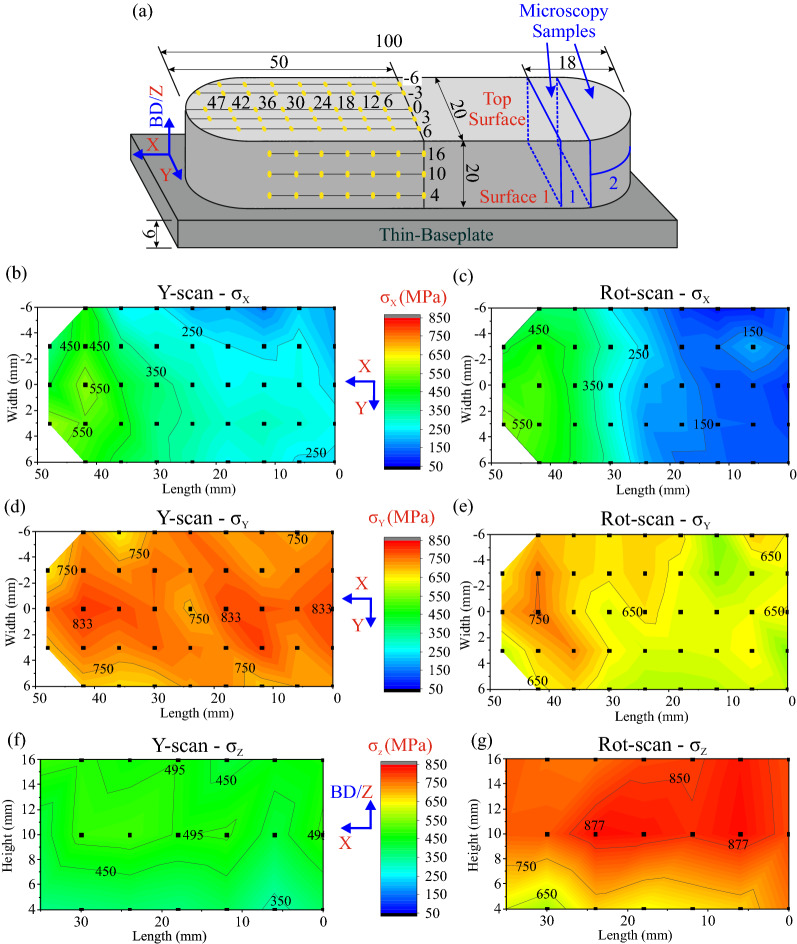
(**a**) Geometry of the SXEDD investigated samples where the location of the measurement points is indicated with yellow dots. (**b**) X-direction (σ_X_) stress maps of the in the Top Surface for the Y-scan and (**c**) Rot-scan samples. (**d**) Y-direction (σ_Y_) stress maps in the Top Surface for the Y-scan and (**e**) Rot-scan samples. (**f**) Z-direction (σ_Z_) stress maps in Surface 1 for Y-scan and (**g**) Rot-scan samples. All the stress maps correspond to the Thin-baseplate condition and their average error is ± 33 MPa.

The location of the near-surface Synchrotron X-ray Energy Dispersive Diffraction (SXEDD) measurement points is shown in Fig. 3a. Assuming geometrical symmetry of the RS fields, the SXEDD measurements were performed on only one half of the sample length. Details about the SXEDD measurements and the RS calculations are given in the Material and Methods section. The X-direction (σ_X_) and Y-direction (σ_Y_) stress components were investigated on the Top Surface, while only the Z-direction (σ_Z_) stress component was investigated on one of the lateral surfaces (labelled as Surface 1).

No significant differences are observed when comparing σ_X_ stress maps of the two samples (see Fig. 3b,c), which is in good agreement with the LXADD results shown in Fig. 2b. The two maps exhibit similar stress distribution with the highest stress occurring at the tip (550–450 MPa). Nonetheless, the stress values in the region between 0 and 30 mm are slightly lower (by 100 MPa) in the Rot-scan sample.

The σ_Y_ stress values are about 200 MPa higher in the Y-scan than in the Rot-scan sample (see Fig. 3d,e). Moreover, the maximum stress values of the σ_Y_ component also occur at the tip of the samples.

Opposite to the above σ_X_ and σ_Y_ components tendency, the σ_Z_ stress maps on Surface 1 indicate considerable higher RS (~ 400 MPa) occurring in the Rot-scan sample (see Fig. 3f,g, where the lowest RS values occur in the bottom region, next to the baseplate). It is noteworthy to recall that the RS values are slightly higher (+ 80 MPa) than the nominal yield strength of the As-Built condition: σ_y//BD_ = 632 MPa when parallel to the BD-axis and σ_y┴BD_ = 806 MPa for the perpendicular one. These values were provided by the material supplier (see section Materials and Methods) and are in good agreement with the values reported in the literature^33,34^.

## Discussion

High levels of RS, nearing the alloy yield strength at room temperature, has been also reported in the literature^5,17,35^. In the present study, it could be argued that the RS values higher than the yield strength result from plastic deformation that brings the material far into the strain hardening regime, thereby increasing the actual yield limit. Since the maximum penetration depth of the SXEDD measurements for the {311} is ~ 42 µm, an assumption of a plane stress state (σ_normal_ = 0) is considered to apply in this study. Nevertheless, a residual stress state at a level higher than the monotonic yield strength could arise from the presence of a triaxial stress state. Hence, the possibility of a triaxial state (e.g., induced by the presence of the theoretically harder micro-segregated phase present at the cell walls) cannot be dismissed and needs further investigation.

It is evident that the choice of scanning strategy has a significant effect on the magnitude and direction of RS. There is controversy in the literature about the direction along which the largest RS magnitudes are build-up. Some authors^5^ state that the stresses perpendicular to the scanning direction (case of scanning strategies using unidirectional vectors) are significantly larger than the stresses along the scanning direction, while others^14,22^ observe that the largest RS occurs parallel to the scanning vectors. In our case, the LXADD results of Fig. 2b,c indicate that the highest values occur in the Y-direction, with the two scanning strategies exhibiting similar values.

Rotational scanning strategies are generally advised for RS mitigation^11,15,23,36^. It is stated that rotation scanning strategies increase the uniformity of stress distribution in As-Built components. The angle of 67° is considered one of the most suitable rotation angles since the scan vector direction does not repeat (considering 10° error) for a large number of layers. However, no significant differences in the uniformity of the stress distributions of Y-scan and Rot-scan samples are observed in the plots of Fig. 2 and the stress maps of Fig. 3. Regarding the minimization of RS, it is observed that the X-direction component follows the commonly reported trend of lower magnitudes for the Rot-scan sample, the Y-direction component exhibits similar values for the two scanning strategies in the As-Built condition while the Rot-scan sample undergoes a higher relaxation after thinning, and the Z-direction component unexpectedly reverses the tendency.

The mechanism leading to the lower σ_Z_ values observed in the Y-scan sample can be explained as follows: in principle, reducing the temperature gradient (dT/dx) decreases the thermal stress developed during material processing, thereby decreasing RS. Thus, a plausible explanation could be the development of a directional thermal transfer, where the heat flux could be favored along the BD-axis during the Y-scan processing and over the SW/ SD-plane during the Rot-scan processing. This would mean that the thermal gradients are higher across the plane orthogonal to the BD-axis for the Y-scan processing, but also on the lateral surface for the Rot-scan.

Another possibility is that the reduced σ_Z_ values observed in the lateral surface of the Y-scan sample are in fact a result of its crystallographic anisotropy (i.e*.,* columnar grains). Using Transmission Electron Microscopy (TEM), some authors have reported^33,37,38^ high densities of dislocations in IN718 LPBF as-built materials. These dislocations form networks that are most commonly entangled at the cell walls. *Therefore,* w*e believe that RS relaxation induced by dislocation accumulation can indeed be, at least, partially responsible for the unexpected results observed in* Fig. 3*.*

In order to verify this dislocation accumulation hypothesis, EBSD maps were acquired at the centre of the MS1 (see Fig. 3a) to compare the amount of Geometrically Necessary Dislocations (GNDs) in the two studied samples. The EBSD data were subsequently refined using a Kikuchi pattern matching software that enables to resolve Low Angle Grain Boundaries (LAGBs) down to 0.05° (more details about this technique are given in the Materials and Methods section).

**Figure 4:**
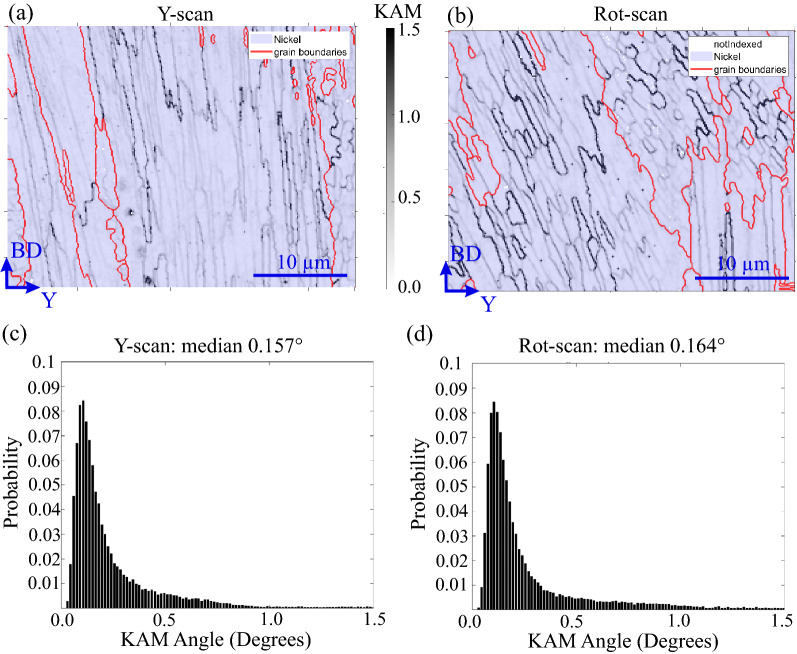
Kernel Average Misorientation (KAM) angle map (step size is 0.15 µm) after Kikuchi pattern matching for (**a**) Y-scan sample and **(b**) Rot-scan sample. The red lines correspond to grain boundaries with misorientations ≥ 2° and the black ones to subgrains with misorientations ≤ 2°. (**c**),(**d**) KAM-angle distributions corresponding to the EBSD maps in (a) and (b), respectively.

Figure 4a shows a high magnification refined Kernel Average Misorientation angle (KAM)^39^ map of the Y-scan sample. Grain boundary angles > 2° are displayed as red lines whereas local orientation changes < 2° are shown in black. When comparing with Fig. 1b, the LAGBs seem to correspond to subgrains formed by columnar dendrite-cells, which exhibit different degrees of misorientation (the most pronounced misorientations correspond to 1.5°). On the other hand, the subgrains observed in the Rot-scan sample (Fig. 4b) alternate mosaic and columnar dendritic-cell microstructures. KAM angle distributions (Fig. 4c,d) are used to compare the character of this misorientation. Despite the different dendritic-cell morphologies, the two materials exhibit similar distributions.

Unfortunately, although EBSD maps with high spatial resolution are suitable for the imaging of subgrain structures, their local nature does not enable a clear comparison between the two scanning strategies. Hence, we also performed EBSD analyses at an intermediate magnification that offers a reasonable sampling of grains, while still being able to partially resolve the subgrain structures.

**Figure 5:**
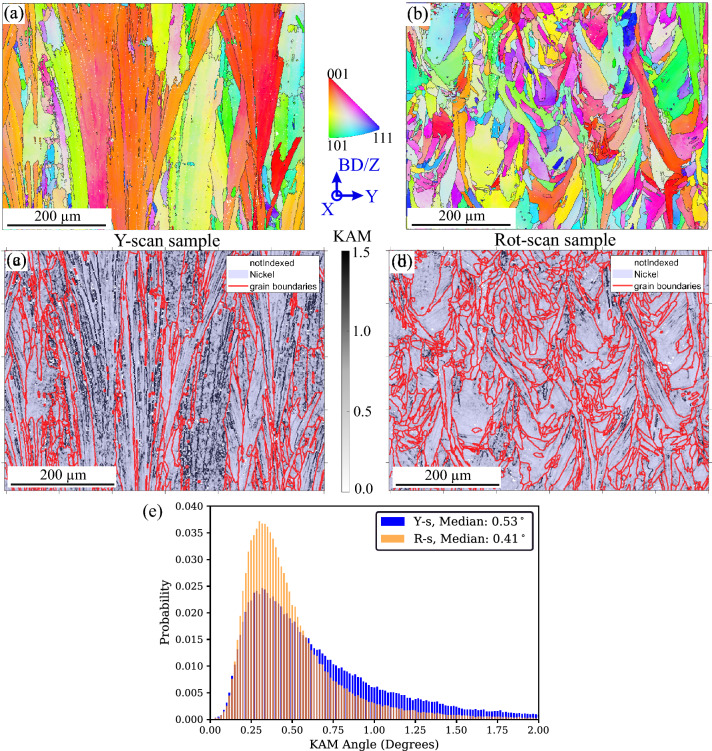
Intermediate magnification EBSD orientation maps for the (**a**) Y-scan sample and (**b**) Rot-scan sample. (**c**) KAM-angle maps from orientation data refined by Kikuchi pattern matching for the Y-scan sample and (**d**) Rot-scan sample. The red lines correspond to grain boundaries with misorientations ≥ 2° and the black ones to subgrains with misorientations ≤ 2°. (e) KAM-angle distributions corresponding to the images shown in (**c**) and (d).

Figure 5a shows an intermediate magnification EBSD orientation map acquired in the Y-scan sample. The Y-scanning strategy leads to the formation of coarse columnar grains (average width of ~ 100 µm), which lay parallel to the BD-axis, span across several layers and exhibit predominance of orientations close to {100}. Instead, the Rot-scan condition (Fig. 5b) yields smaller grains that exhibit elongated shapes. Such grains seemingly span across a lesser number of layers and seem to grow in random directions, so that no dominant crystallographic orientation is observed. The difference in the amount of KAM between the two scanning strategies is already noticeable from a qualitative standpoint (Fig. 5c,d), with the Y-scan sample exhibiting higher levels of subgrain boundaries. Note that the degree of misorientation seems to depend on the grain orientation, as some of the grains show larger populations of subgrains, especially in the Y-scan sample. The corresponding KAM distributions in Fig. 5e show a compensation between decreased population of very low-angle boundaries (< 0.5°) and increased population of low angle boundaries (0.75° to 2°) when comparing the Y-scan sample to the Rot-scan sample. An increase in the amount of high-angle boundaries with persistence of low-angle boundaries is usually related in the literature to deformation-induced microstructures^39^.

**Figure 6:**
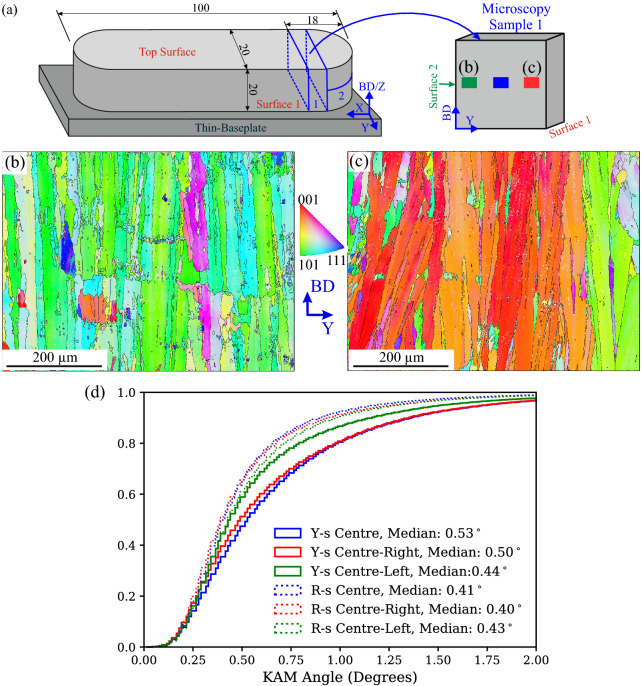
(**a**) Schematic illustration of the location of the MS1, with a detail showing the location of the additional EBSD maps. (**b**) Intermediate magnification EBSD orientation maps for the Y-scan sample corresponding to the Centre-Left region. (**c**) Intermediate magnification EBSD orientation maps for the Y-scan sample corresponding to the Centre-Right region. (d) KAM-angle cumulated distribution showing the results from all six EBSD maps.

In order to get an idea about the reproducibility of the results shown in Fig. 5, we mapped in each sample two additional, equal-sized areas under comparable conditions. Their locations are sketched in Fig. 6a, and the resulting orientation maps corresponding to the Y-scan sample are displayed in Fig. 6b,c. Interestingly, the EBSD map corresponding to the Centre-Left region (centre of this EBSD map lays at 3 mm from the free surface) exhibits a clear color change while the ESBD map of the Centre-Right region expresses a crystal orientation tendency similar to the one observed at the centre of the sample (Fig. 4a). The cumulative frequency of the KAM distributions of all six EBSD maps is shown in Fig. 6d. Irrespectively of the location, the Rot-scan sample possesses comparable KAM distributions. On the other hand, the Y-scan sample exhibits an increased inhomogeneity, where the Centre-Left region contains lower amounts of misorientation compared to the Centre and Centre-Right regions. It must be noted that the growth of large columnar grains in the Y-scan sample needs to compensate any defects during solidification by misorientations. This can result in a higher defect density and stronger misorientations. Moreover, even though the amount of grains sampled in the EBSD maps is considerable, it is still not enough to obtain statistically significant results of the studied samples. Compared to the Rot-scan sample, the increased spread of data observed in the Y-scan sample could be as well partially induced by the lower amount of grains sampled by the EBSD maps.

Keeping in mind the aforementioned statistics limitations and the local nature of the EBSD results, we consider that Fig. 6d gives indication about the fact that the unexpected lower σ_Z_ values observed in the Y-scan sample (Fig. 3g,h) are very likely influenced by the underlaying microstructure. Large columnar grains would favor the relaxation of σ_Z_ component by an increased dislocation accumulation. The higher levels of misorientation observed in the centre and Surface 1 regions of Y-scan sample would also suggest that favorably oriented columnar grains are able to accumulate more dislocations.

**Figure 7:**
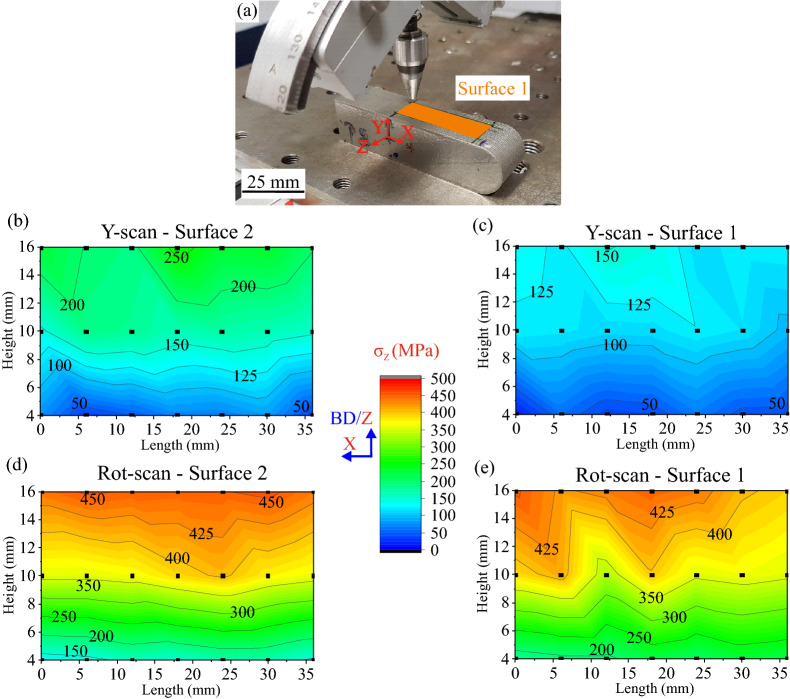
(**a**) LXADD setup corresponding to the RS investigation of the Released Rot-scan sample (**after** baseplate removal). (**b**),(**c**) Maps of the Z-direction RS component in the Y-scan sample on Surface 2 and Surface 1, respectively. (**d**),(**e**) Maps of the RS Z-direction component in the Rot-scan sample on Surface 2 and Surface 1, respectively. The average error is ± 20 MPa.

Instead of proceeding with further laborious EBSD examination to increase the statistics of Fig. 6d, or even to perform High Resolution Synchrotron Diffraction (HRSD) for the measurement of dislocation structures^40^; the possible influence of the microstructure was re-examined on the lateral surfaces of both samples: *after* release from the baseplate (i.e*.,* Released condition) we performed RS measurements using LXADD (see Fig. 7a).

For the two samples, the RS levels are quite similar in the bottom-half, while higher RS values are observed in the upper-half (Fig. 7b,e). However, higher RS occur in Surface 2 compared to Surface 1 for the Y-scan sample, while no significant differences are observed between the two surfaces of the Rot-scan sample. The latter finding is in good agreement with the microstructure homogeneity shown in Fig. 6d. Also, the RS state is always lower in the Y-scan sample when compared to the Rot-scan one. A possible explanation for the results of Fig. 7 would be that that the magnitude of the Z-direction RS component in the Y-scan sample is reduced by dislocation accumulation, and that this accumulation is mainly driven by larger grain sizes and favorable crystallographic orientations. The spatially dependent heterogeneity of Y-scan sample would enable the accumulation of higher amounts of dislocations in one part of the sample (adjacent to Surface 1), therefore leading to the lower RS values observed in Fig. 7c.

A considerable reduction of the Z-direction RS values is observed between the SXEDD Thin-Baseplate (Fig. 3f,g) and LXADD Released results (Fig. 7c,e). This reduction is likely induced by stress relaxation after baseplate removal (mostly occurring at the bottom), but also by the fact that LXADD and SXEDD techniques do not interrogate the same penetration depth (~ 5 µm for LXADD and ~ 42 µm for SXEDD analysis).

Our rational to correlate the bulk EBSD analysis to SXEDD measurements in the near-surface is based on the principle of RS balance, whereby the near surface regions equilibrate the bulk residual stress state. Thus, any RS relaxation occurring in the bulk material should reflect on the near-surface RS state^41^. It must be noted that a deeper understanding of microstructure-induced RS relaxation needs the use of monochromatic Neutron Diffraction (ND). Nevertheless, strong textured bulk microstructures such as the one observed in the Y-scan samples hampers the use of this technique (e.g., vanishing of the reflection peak for certain directions). Further work needs to be done to overcome the challenges of residual stress analysis in highly textured materials both with monochromatic and energy dispersive (time-of-flight) neutron diffraction.

**Figure 8:**
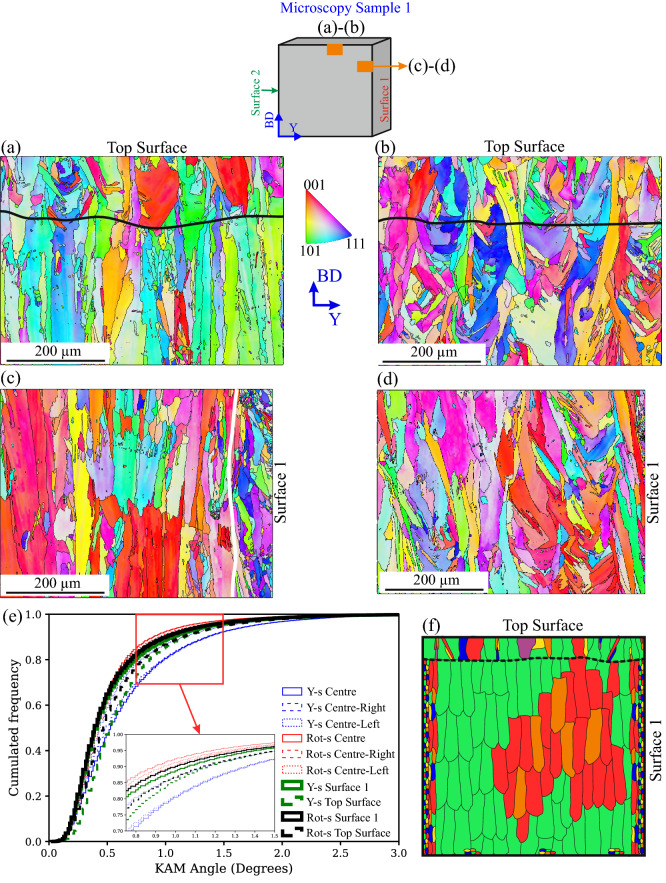
EBSD orientation maps corresponding to the subsurface region below the Top Surface for (**a**) the Y-scan sample and (**b**) the Rot-scan sample; the black horizontal lines indicate the approximate location of the border between the bulk material and the up-skin layer. EBSD orientation maps corresponding to the subsurface region below Surface 1 for (**c**) the Y-scan sample (where white line indicates the approximate location of the transition between fine grains and larger and more textured grains) and (**d**) the Rot-scan sample. (**e**) Plot of the cumulated frequency against KAM angle, where the data that have been already introduced in Fig. 6d is shown is blue and red, and the data corresponding to the subsurface regions is shown in green and black. (**f**) Schematic illustration summarizing the main findings of the EBSD analysis in the Y-scan sample.

**Figure 9:**
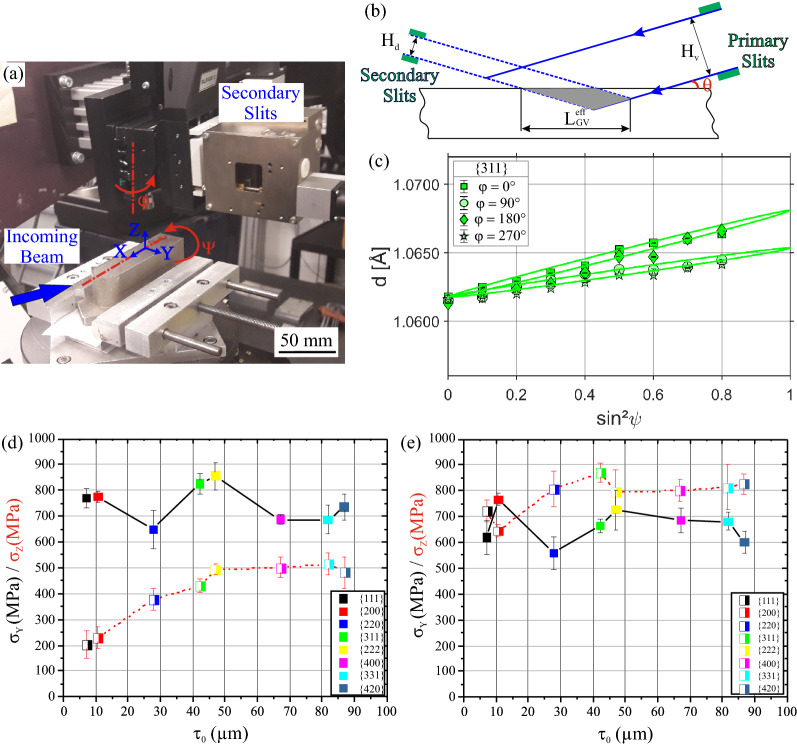
(**a**) The synchrotron experimental set-up corresponding to the analysis of the Y-component on the Top Surface, where φ = 0° and $\uppsi$ = 0°. (**b**) Schematic illustration of the gauge volume immersion, where the information depth area is shown in grey. (**c**) ${\mathrm{d}}_{\uppsi }^{\mathrm{hkl}}-{\mathrm{sin}}^{2}{\uppsi}$ distribution showing the linear regression without $\uppsi$-splitting of a point measured on the Top Surface of the Y-scan sample (**d**) Plot of the Y and Z-direction stress values obtained by linear regression from ${\mathrm{d}}_{\uppsi }^{\mathrm{hkl}}-{\mathrm{sin}}^{2}{\uppsi}$ distributions. The stress is plotted versus the maximum information depth τ_0_, corresponding to a point in the Top Surface (black solid line) and a point in Surface 1 (red dashed line) of the Y-scan sample. (**e**) Plot of the Y and Z-direction stress versus τ_0_ corresponding to a point in the Top Surface (black solid line) and a point in Surface 1 (red dashed line) of the Rot-scan sample.

The reason why it was possible to obtain linear lattice-spacing-vs-sin^2^ $\uppsi$ distributions with reduced oscillations during the LXADD and SXEDD measurements (see Fig. 9c in the Material and Methods section) is because the near-surface grain structure is finer with reduced texture. As shown in Fig. 8a, the manufacturing of the up-skin layer in the Y-scan sample locally halts the epitaxial growth characteristic of the bulk material, promoting the growth of grains that span through the height of the up-skin layer. The transition between the bulk material and the up-skin layer is, however, less noticeable in the Rot-scan sample (Fig. 8b). Interestingly, the KAM distribution results of these two regions (Fig. 8e, green and black dashed lines) are shifted rightwards with respect to the bulk results. The KAM angle maps (not shown in Fig. 8 for the sake of brevity) indicate small regions of increased misorientation occurring at the border between the bulk and the up-skin; it is suggested that the processing of the up-skin layer leads to a local increase of the misorientation content in both samples.

Figure 8c shows that a subsurface layer of ~ 100 µm containing finer grains is formed underneath Surface 1 of the Y-scan sample (the approximate border is shown with a white vertical line). From this depth inwards, the microstructure exhibits columnar grains, which, in general, are smaller than those observed in the bulk, and where <001>-orientation is dominant. In the case of the Rot-scan sample (Fig. 8d), and apart from a thin (~ 50 µm) layer of fine grains, the microstructure beneath Surface 1 is considered similar to that observed beneath the up-skin (Fig. 8b) and the bulk (Fig. 5b). The KAM evaluation (Fig. 8e, green and black solid lines) indicates similar results for these two regions of the Y-scan and Rot-scan samples, with the misorientation values laying between the bulk values of Rot-scan sample and the Centre-left values of the Y-scan sample.

The clarification of the KAM differences observed between the subsurface regions of Fig. 8 requires further investigation. In any case, we think that these differences are mainly driven by the processing itself and/or the local microstructure. The RS relaxation (leading to an increased misorientation content) would mainly occur in the bulk of Y-scan sample, where the conditions of large textured columnar grains are fulfilled (see Fig. 8f). Moreover, because of preferred orientation, regions with <001> dominance would be able to accumulate higher amounts of misorientation, therefore increasing the RS relaxation effect.

Overall, it is observed that an EBSD analysis of the material, even when intensively performed, might not suffice for a complete characterization of spatially dependent microstructures characteristic of AM materials, emphasizing the need for the use of complementary techniques such as texture analysis by XRD/ND and Bragg-edge tomography^42^.

## Conclusions

The as-built RS state of LPBF IN718 samples manufactured using two different scanning strategies was examined by means of X-ray diffraction. The use of EBSD data refined by Kikuchi pattern matching permitted for an insight to be gained into the grain structure and KAM of the materials. The results presented in this study indicate that the effect of scanning strategies need to be evaluated not only in terms of thermal stress mechanisms, but also considering the influence of the resulting microstructure. Nonetheless, the spatially dependent microstructural heterogeneity in combination with the strong texture observed in the Y-scan sample poses a major challenge for both microstructural and bulk RS characterization. These challenges need to be addressed for the further advancement of AM materials with strong textures.

It is also observed that the underlaying grain-structure could influence the amount of RS relaxation induced by dislocation accumulation. Since materials which are precipitation hardened during post building heat treatments show sensitivity to the as-built microstructure, the process-induced variability in dislocation content should also be considered when envisaging postprocess thermal treatments^34,43,44^.

## Material and methods

### LPBF

The samples were manufactured by Siemens AG, Power and Gas Division, Berlin, Germany, using an EOS M290 machine. The prealloyed IN718 powder was produced by gas atomization. The geometry of the specimens is given in Fig. 1a. The studied samples were produced in the same build job. Further details on the manufacturing parameters cannot be disclosed as they are proprietary to Siemens AG. In order to obtain a fine surface finishing, different manufacturing and scanning parameters were used for the manufacturing of the last three top layers (named as the up-skin layer). Figure 1b,c show details of the scanning strategies used to produce the Y-scan and Rot-scan samples.

### SEM-EBSD analysis

The Microscopy Samples (MS) were cut out of one end of the specimens using Electro-Discharge Machining (EDM, see Fig. 1a). LXADD (the results are not reported in this work) was used to confirm that RS in the investigated region remained unaltered after the removing of MS^45^. MS1 was used for analysis of the BD-Y plane and MS2 for the X-Y plane. Metallographic preparation consisted of grinding with SiC P120, P320, P600 and P1200-grit papers. Polishing was performed using 6, 3 and 1 µm diamond particle suspensions and 0.02 µm non-crystallizing colloidal silica suspension for the finishing. The MS1 samples required up to 30 min of finishing to avoid any scratch appearing on the refined KAM-angle maps. A LEO 1530VP (ZEISS) SEM equipped with an EBSD system from Bruker was used for the microstructural characterization. The EBSD data were acquired with an ESPRIT 1.94 (Bruker Nano) system. The step size was 0.15 µm in the high magnification maps and 1.2 µm in the intermediate magnification ones. The Kikuchi pattern matching used the rough orientation description delivered by the EBSD system, and a subsequent cross correlation of the stored Kikuchi patterns with simulated patterns was applied to improve the orientation correctness. The computing times for the matching method are considerably higher than the acquisition of the EBSD data, limiting the possibility of its systematic use. For further details on the pattern matching, the reader is referred to^46^.

### SXEDD measurements

The Synchrotron X-ray Energy Dispersive Diffraction results were acquired at the Energy Dispersive Diffraction (EDDI) beamline^47^, located in the BESSY II synchrotron (Helmholtz Zentrum Berlin (HZB), Germany). This beamline operates in energy-dispersive mode, providing a white beam with a 10 to 150 keV energy range. The diffraction experimental setup is shown in Fig. 9a. The diffraction angle was set at 2 $\uptheta$ = 10° and kept constant in all measurements. The sin^2^ $\uppsi$ -method^48^ is used for RS determination (nine $\uppsi$ angles in total). The location of the measurement points is given in Fig. 3a.

The SXEDD analysis has the advantage of obtaining the lattice spacing d^hkl^ for different crystallographic reflections *hkl* as a function of the energy *E*_hkl_: ${d}^{hkl}=\frac{6.199}{sin\theta }\frac{1}{{E}_{hkl}(keV)}$. The white beam enables the probing of different depths^49^, with the penetration depth τ being determined by^47^: $\uptau = \frac{sin\theta }{2\upmu ({\mathrm{E}}_{hkl})}{cos\psi }$; where µ(*E*_hkl_) is the linear absorption coefficient at the corresponding *E*_hkl_ energy level. In the present work, the depth resolution is based on the Laplace methods^50^. Typically, the stresses obtained from different interference lines *hkl* are plotted against an average information depth ($\langle {\uptau }^{\mathrm{hkl}}\rangle = ({\uptau }_{0}^{\mathrm{hkl}}+ {\uptau }_{\mathrm{max}}^{\mathrm{hkl}})$), where ${\uptau }_{0}^{\mathrm{hkl}}$ is the maximum information depth at $\uppsi$ = 0° and ${\uptau }_{\mathrm{max}}^{\mathrm{hkl}}$ is the maximum information depth at $\uppsi$ = max (in this study the max corresponds to 63°). Nevertheless, it has been recently reported^51^ that plotting the sin^2^ $\uppsi$ data versus the maximum information depth ${\uptau }_{0}^{\mathrm{hkl}}$ results in a discrete depth distribution that coincides with the actual Laplace space stress depth profile σ(τ). Therefore, the maximum information depth ${\uptau }_{0}^{\mathrm{hkl}}$ has been adopted in the current study, where the depth values for the {311}-reflection correspond to ${\uptau }_{0}^{\mathrm{hkl}}$= 42 µm instead of $\langle {\uptau }^{\mathrm{hkl}}\rangle =$ 33 µm.

The gauge volume geometry is defined by the intersection of the incoming beam (with vertical and horizontal openings of H_v_ = H_h_ = 1 mm) and the diffracted beam (the secondary slits had a vertical opening of H_d_ = 30 µm, see Fig. 9a,b). The effective gauge volume length was ${\mathrm{L}}_{\mathrm{GV}}^{\mathrm{eff}}$ = 5.9 mm. Nevertheless, the information depth (i.e*.*, the interrogated region, shown in grey in Fig. 9b) is usually smaller. In order to calculate it, the exponential beam attenuation and the geometrical shape of the gauge volume need to be considered^52^.

The X- and Y-direction components were measured by rotating the sample at two φ positions (φ = 0° for Y-direction and φ = 90° for X-direction). A CCD camera and a laser triangulation system are used to determine the measuring position on the sample surface. Since measurements were performed in the reflection mode, an in-plane biaxial stress field can be assumed and thereby the stress component normal to the sample surfaces can be considered zero (corresponding to the Z-direction in the Top Surface and the Y-direction in Surface 1). The linear regression of the ${\mathrm{d}}_{\uppsi }^{\mathrm{hkl}}-{\mathrm{sin}}^{2}\uppsi$ distribution allows to obtain the in-plane RS values corresponding to each measured φ angle (Fig. 9c shows the {311}-reflection). The error of the linear regression is calculated using the error propagation equation:

$$er{r}_{\sigma \left(hkl\right)}= \frac{1}{{d}_{0}({\mathrm{hkl}})}* \frac{1}{0.5*{S}_{2}^{hkl}}*{err}_{slope(hkl)}$$

where *d*_*0*_(hkl) is the spacing of the unstressed lattice.

Assuming that X, Y and Z are the principal stress directions, the fundamental equations of X-Ray stress analysis for φ = 0° and φ = 90° become ^41^:

$$\begin{aligned} \varepsilon_{(0,\psi )} & =1/2S_{2} \sigma_{Y} {\sin}^{2} \psi + S_{1} (\sigma_{X} + \sigma_{Y} )\,  \qquad {\text{and}} \\ \varepsilon_{(90,\psi )} & =1/2S_{2} \sigma_{X} {\sin}^{2} \psi + S_{1} (\sigma_{X} + \sigma_{Y} ). \\ \end{aligned}$$

where *S*_*1*_ and *S*_*2*_ are the Diffraction Elastic Constants (DEC). Table 1 summarizes the Reuss DEC^53^ used in this study.

**Table 1:** The Reuss DEC for several crystallographic reflections of Ni.

hkl	S_1_, MPa^-1^ × 10^–6^	½S_2_, MPa^-1^ × 10^–6^
111	− 0.763	4.1
200	− 2.94	10.6
220	− 1.31	5.73
311	− 1.92	7.56
400	− 2.94	10.6
331	− 1.15	5.26
420	− 1.9	7.5

The EDDIDAT program system^54^ provided by the X-ray CoreLab (HZB) was used for handling of measurement data and calculating the results. A Pseudo-Voigt function is used for the peak fitting. The lattice spacing against sin^2^ $\uppsi$ plots show insignificant $\uppsi$-splitting (an example is given in Fig. 9c) and, therefore, the shear stresses are considered negligible. Owing to the finer grain-structure occurring in the near-surface region (Fig. 8), the plots show a linear relationship between the {311}-lattice strain and sin^2^ $\uppsi$. The choice of {311} as the diffracting reflection was made following the recommendations of the ISO 21432:2019 standard^55^.

The surface roughness was evaluated according to DIN EN ISO 4287:2010 standard^56^ using a Hommel Werke TKL300 system equipped with a tip of 5 µm radius. The calculated roughness values are presented using the R_a_ (arithmetic mean height) and R_z_ (maximum height) paremeters. The measurements on the Top Surface of the Y-scan sample give R_a_ = 4.08 ± 0.53 µm and R_z_ = 22.03 ± 3.84 µm. As a result of the up-skin processing, the Top Surface roughness values of the Rot-scan sample are considered similar to those of the Y-scan sample. The roughness on Surface 1 and Surface 2 of the Rot-scan sample is R_a_ = 4.52 ± 1.12 µm and R_z_ = 31.83 ± 9.78 µm, while the roughness on Surface 1 and Surface 2 of the Y-scan sample increases to R_a_ = 11.75 ± 0.75 µm and R_z_ = 72.8 ± 6.39 µm.

Examples of the stress versus the maximum information depth τ_0_ relation are given in Fig. 9d, e. On the Top Surface (points connected with black solid lines), the RS values display an overall relatively flat distribution within the scatter with depth, with σ_Y_ variations between 650 and 850 MPa for the Y-scan sample and between 575 and 750 MPa for the Rot-scan sample. The effect of surface roughness, which is attributed to a lower magnitude of residual stress, is clearly observed in Surface 1 of the Y-scan sample (Fig. 9d, red dashed line), where the σ_z_ values exhibit a linear increase as a function of depth until a plateau is reached between {311} and {222} reflections at τ_0_ = 45 µm. From this depth up to {420} reflection, the σ_z_ values remains stable at 500 MPa. The effect of the surface roughness is less noticeable in Surface 1 of the Rot-scan sample, where σ_z_ increases as a function of depth until the depth of the {220} reflection. In this case, the plateau starts from τ_0_ = 30 µm and σ_z_ values vary between 800 and 900 MPa. These results are in good agreement with a previous study by the authors, where the impact of surface roughness was investigated using a LPBF Ti6Al4V material^57^. In this previous study, the effect of surface roughness on the relaxation of RS is also reported to vanish once the plateau is reached.

### LXADD measurements

Laboratory Angular Dispersive X-ray Diffraction was performed using a Xstress G3 diffractometer (StressTech, Vaajakoski, Finland). This type of the diffractometer is equipped with a movable arm carrying an X-ray source and two position sensitive detectors that revolve around a stationary sample. A MnKα radiation source was used with a collimator aperture of 2 mm in diameter. The penetration depth of the laboratory radiation is estimated to be ~ 5 µm. The sin^2^ $\uppsi$ -method was also used for RS determination. The Ni-311 reflection (2θ = 156°) was measured in an angular range from ψ = − 45° to ψ = 45° with a total of 19 steps. The Reuss evaluation parameters used in LXADD are the same as those used in the SXEDD data. The peak fitting was performed using the Pearson VII function.

## Data Availability

The datasets generated and/or analysed for the present study are available upon reasonable request to the corresponding author.
